# Pancreatic cancer stemness: dynamic status in malignant progression

**DOI:** 10.1186/s13046-023-02693-2

**Published:** 2023-05-13

**Authors:** Yutong Zhao, Cheng Qin, Bangbo Zhao, Yuanyang Wang, Zeru Li, Tianyu Li, Xiaoying Yang, Weibin Wang

**Affiliations:** 1 Department of General Surgery, Peking Union Medical College Hospital, Peking Union Medical College, Chinese Academy of Medical Sciences, Beijing, 100023 People’s Republic of China; 2 Key Laboratory of Research in Pancreatic Tumor, Chinese Academy of Medical Sciences, Beijing, 100023 People’s Republic of China; 3 National Science and Technology Key Infrastructure On Translational Medicine in, Peking Union Medical College Hospital, Beijing, 100023 People’s Republic of China

**Keywords:** Pancreatic cancer, Cancer stem cells, Stemness, Tumor microenvironment, Plasticity

## Abstract

Pancreatic cancer (PC) is one of the most aggressive malignancies worldwide. Increasing evidence suggests that the capacity for self-renewal, proliferation, and differentiation of pancreatic cancer stem cells (PCSCs) contribute to major challenges with current PC therapies, causing metastasis and therapeutic resistance, leading to recurrence and death in patients. The concept that PCSCs are characterized by their high plasticity and self-renewal capacities is central to this review. We focused specifically on the regulation of PCSCs, such as stemness-related signaling pathways, stimuli in tumor cells and the tumor microenvironment (TME), as well as the development of innovative stemness-targeted therapies. Understanding the biological behavior of PCSCs with plasticity and the molecular mechanisms regulating PC stemness will help to identify new treatment strategies to treat this horrible disease.

## Introduction & background

Pancreatic cancer (PC) represents a major cause of cancer-related death worldwide and differs from other cancers, the incidence rate of PC patients has continued to increase over the past few years, with little improvement in survival rates [1]. Alarmingly, PC has a poor 5-year survival rate, less than 11% in the US [2], and it has been projected that the mortality of PC will become the second leading cause of cancer-related deaths in the US by 2030 [3].

Many tumor entities have made considerable advances in the diagnosis and treatment during the past decade; however, this is not the case for PC. From a clinical point of view, the comparatively low success rate of therapy for PC compared with other cancers is attributable to the deep location of the pancreas causing a lack of appropriate screening and diagnostic modalities and challenges in performing a tissue biopsy, aggressive clinical course, and low response rate of PC to chemo- and radiotherapy [4]. From the characteristics of the PC itself, in addition to the highly heterogeneous tumor immunosuppressive microenvironment, PC stem cells (PCSCs) are also functionally important in tumor progression and therapeutic resistance [5]. Tumors are comprised of a limited number of distinct cells known as cancer stem cells (CSCs), also referred to as tumor-initiating cells (TICs). They possess the ability of tumorigenesis reconstitution with unlimited proliferative potential and inherently higher chemo- and radioresistance, have increased metastatic and invasive potential and show higher disease recurrence compared with their differentiated cancer cell counterparts [5, 6]. Therefore, a deeper understanding of CSCs is necessary for the improved management of cancer patients.

The presence of CSCs was first demonstrated in acute myelogenous leukemia in 1994 [7, 8] and subsequently confirmed in breast [9] and brain tumors [10]. Three different studies using mouse models of the brain, skin, and intestine gave the first convincing data to demonstrate the involvement of CSCs in malignancies progression [11–13]. PCSCs were first identified in 2007 and represent less than 1% of all PC cells [14]. These cells were first discovered as CD44 + CD24 + ESA + cells with the ability to develop tumors at a significantly higher frequency than the bulk tumor [14]. However, later studies have shown that PCSCs can express multiple markers, including CD9, CD24, CD34, CD44, CD133, ABCB1, ABCG2, ALDH1, CXCR4, DCLK-1, ESA, EZH2, GLRX3, NANOG, OCT4, SOX2, NOTCH-1, c-MET, LGR5, alpha6beta4, tetraspanin-8 and nestin [15–20]. The expression of these factors reprograms cells to CSCs and promotes plasticity, thereby allowing tumor cells to adapt to changes in their environment and survive. Furthermore, the interactions and connections among CSC markers in PC are quite complicated. Specifically, these markers are poor prognostic indicators linked to tumor clinical progression and recurrence. CSCs promote tumorigenesis, chemical resistance and metastasis. Theoretically, eliminating CSCs may be a promising approach for PC treatment. However, an increasing number of studies have shown that CSCs exhibit strong plasticity and this plasticity allows them to be successfully adapted to targeted therapies [21, 22]. In 2008, Patrick C. Hermann et al. first proposed that PCSCs are in a plastic state rather than a hardwired defined state [23]. In general, the plasticity of CSCs can be defined as the ability of cells to differentiate across lineages and hierarchies and refers to the ability of cancer cells to generate more differentiated bulk tumor cells, as well as cells’ phenotypic potential——the capacity of cells to adopt a new identity or fate in response to changing circumstances and environmental factors, leading to increased tumor heterogeneity and promoting tumor progression [23, 24].

**Fig. 1:**
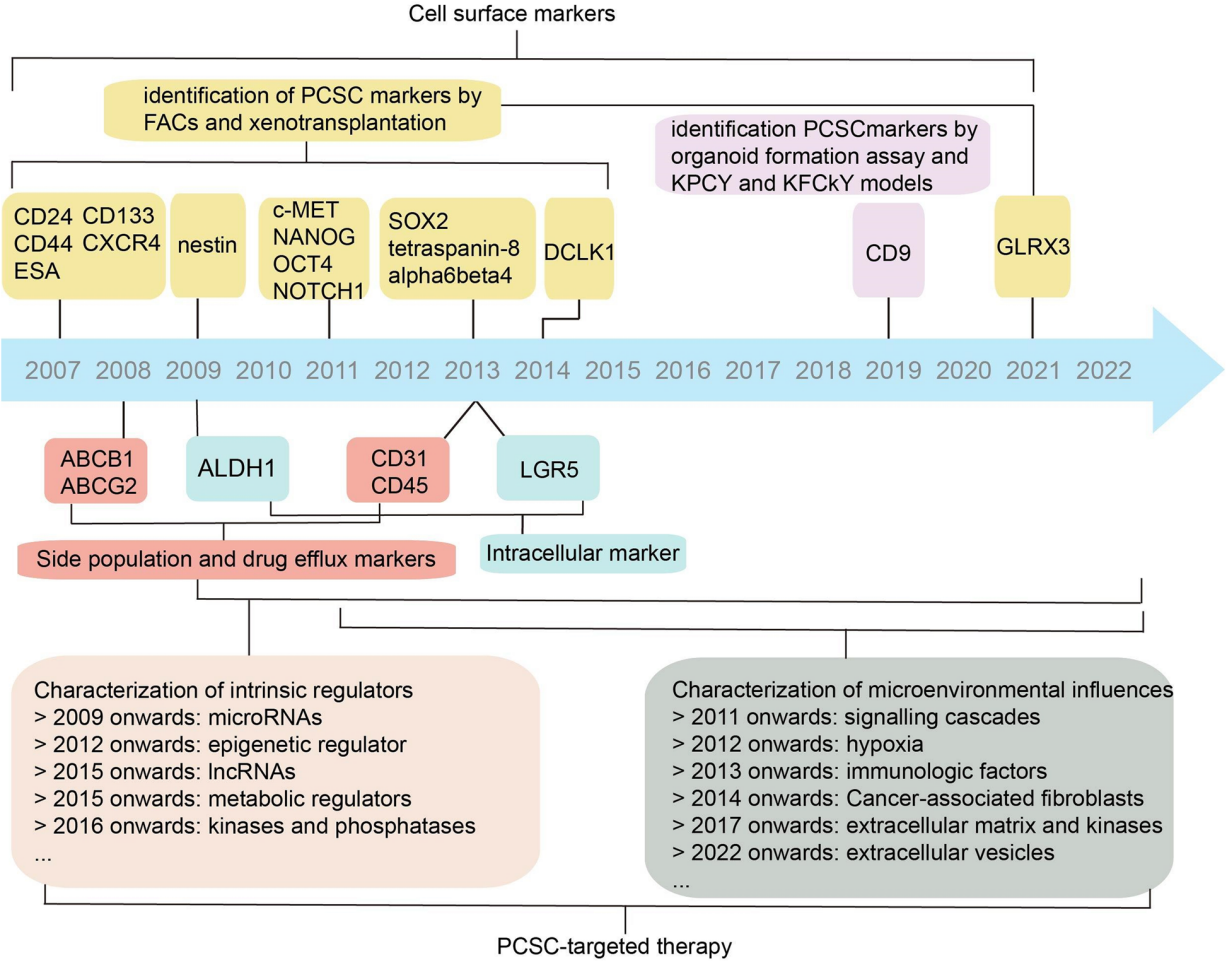
Early studies using FACs and xenotransplantation techniques identified markers of pancreatic cancer stem cells, which laid an important foundation for further research. Following the identification of these markers, a great deal of work has been devoted to exploring the intrinsic and extrinsic regulators of pancreatic cancer stemness

Herein, we summarize current knowledge of PCSCs from an oncology perspective, discuss developments in the field of PCSCs and, more importantly, focus on elucidating that PCSCs exist as a plastic state, influenced by multiple factors inside and outside the tumor cell, influenced by multiple factors inside and outside the tumor cell and, highlight the role of PCSCs in contributing to the malignant behavior of tumor and their potential clinical applications, which provide a comprehensive understanding of the plasticity of PCSCs and its roles in cancer progression. Figure 1 shows the milestones and discovery timeline related to PCSCs.

### Classical markers and signaling pathways of PCSCs

#### Markers

Early research on PCSCs mainly focused on identifying their markers through flow cytometry and xenotransplantation assays in immunocompromised mice. Although it is not completely convincing to rely only on markers to identify PCSCs, these early studies have laid a solid foundation for later research on PC stemness. These markers do not exist independently, instead, interacting with each other and with stemness-related pathways to promote tumor progression. The triplet combination of CD24 + CD44 + ESA + and the binary CD133 + CXCR4 + combination represent the earliest identified PCSC surface markers [25, 26]. In addition to classical cell surface markers, side population and drug efflux markers, as well as intracellular markers have also attracted researchers' attention. High expression of ATP-binding cassette (ABC) transporters was found on the plasma membrane of many CSCs, which is responsible for transporting small molecules from the cytoplasm to the extracellular using the energy generated by ATP hydrolysis. It has been proved that the expression of ABCB1 and ABCG2 in PCSCs is increased [27]. The increase of ABC transporters in CSCs enhances their ability to excrete dyes and drugs. With this characteristic, PCSCs in large tumor samples can be identified by Hoechst 33,342 and flow cytometry. In addition, the enhanced efflux ability of drugs also increases chemotherapy resistance. Different ABC transporters exhibit different efflux abilities for different compounds. For example, ABCG2 mediates resistance to 5-flurorouracil and irinotecan—chemotherapeutic agents for PC [28]. PCSC markers, the associated pathways and their effect on tumor progression are summarized in Table 1.

**Table 1:** Pancreatic cancer stem cell markers, the associated pathways and their effect on tumor progression

Name	Functions on PC and major associated signaling pathways
Cell surface markers	
CD24	JADE dependent AKT/mTOR pathway; SHH pathway
CD44	JADE dependent AKT/mTOR pathway; SHH pathway; SPP1/CD44 pathway; CD44/ITGB1 pathway; Wnt pathway;
ESA/EpCAM	SHH pathway; Wnt pathway
CD133	CCL21/CCR7 axis
CXCR4	SDF-1/CXCR4 axis
nestin	TGFβ/SMAD4 pathway
c-MET	YAP/HIF-1α axis
NANOG	Wnt pathway; NOTCH pathway; SHH pathway
OCT4	SHH pathway; Wnt pathway; NOTCH pathway
NOTCH1	NOTCH1/Jagged1/Hes1 axis
SOX2	FGFR/AKT/SOX2 axis
Tspan8	SHH pathway
α6β4	Form hemidesmosomes
DCLK1	Regulate miRNAs; Histone modification
CD9	Modulate glutamin metabolism
GLRX3	Met/PI3K/AKT; Combine with CA19-9 increase the sensitivity of diagnosis
Side population and drug efflux markers	
ABCB1	Not reported
ABCG2	ERK1/2/HIF-1α axis
CD31	Not reported
CD45	Not reported
Intracellular markers	
ALDH1	Wnt
LGR5	Not reported

### Signaling pathways and targeted therapy

#### Major signaling pathways

**Fig. 2:**
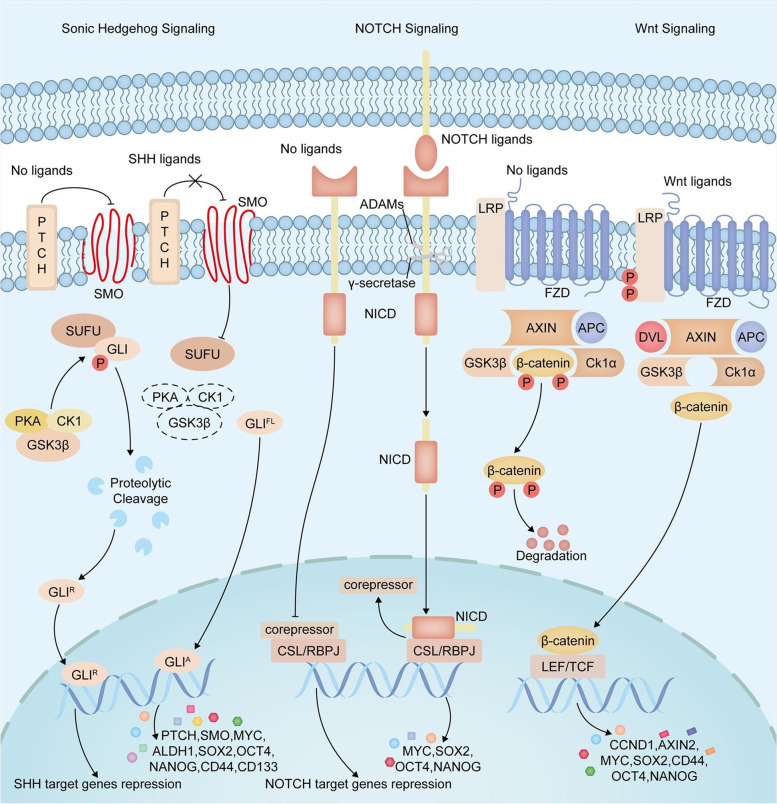
In the absence of ligands, the SHH signaling pathway is inactive. When hedgehog ligands activate the SHH pathway, these hedgehog ligands bind to PTCH and relieve the repressive effects on SMO, a seven-transmembrane protein, resulting in the translocation of the full length activated forms of GLI (GLI^A^) into the nucleus and activation of the expression of target genes, including PTCH and GLI themselves and many other cancer stemness-related genes. When the receptor activates the NOTCH pathway by binding with its ligand, the protein of the receptor is cleaved by a disintegrin and metalloproteinases (ADAMs) that mediate the cleavage of the NOTCH extracellular domain and γ-secretase, which mediates the cleavage of the NOTCH intracellular domain (NICD), which is then released. NICD is further translocated to the nucleus as a transcriptional effector and displaces the corepressor protein from CSL (CBF1, Suppressor of Hairless, LAG1)/RBPJ transcription factors, which leads to the activation of downstream signaling cascades to regulate target genes, including Hes1, Hey1, cyclinD1, c-myc, p21/Waf1 and nuclear factor-κB(NF- κB). WNT ligands are responsible for activating the WNT/β-catenin signaling pathway. In the absence of WNT ligands, β-catenin is phosphorylated by the destruction complex of β-catenin, which is a tertiary complex formed by the scaffolding proteins Axin and adenomatous polyposis coli (APC), the kinase proteins glycogen synthase kinase-3β (GSK-3β) and casein kinase 1α (Ck1α). This leads to β-catenin degradation via β-TrCP200 ubiquitination; therefore, β-catenin is failed to translocate from the cytoplasm to the nucleus. Contrastingly, in the presence of WNT ligands, the ligands bind to the FZD and the LRP receptor complex. Then the cytoplasmic domain of the LRP receptor is phosphorylated by GSK3β and CK1α, and the scaffolding protein Disheveled (Dvl) is recruited. The unphosphorylated β-catenin is released from the complex and enters the nucleus. In the cytoplasm, TAZ/YAP directly interacts with β-catenin and restricts its degradation. In the nucleus, β-catenin binds to TCF/LEF and enhances the recruitment of histone-modifying coactivators, such as BCL9, CBP/p300, and BRG1, to induce the transcription of WNT target genes. Many of these genes encode gene products capable of broadly upregulating cancer cell stemness, including CCND1, AXIN2, and the oncogene MYC

**Fig. 3:**
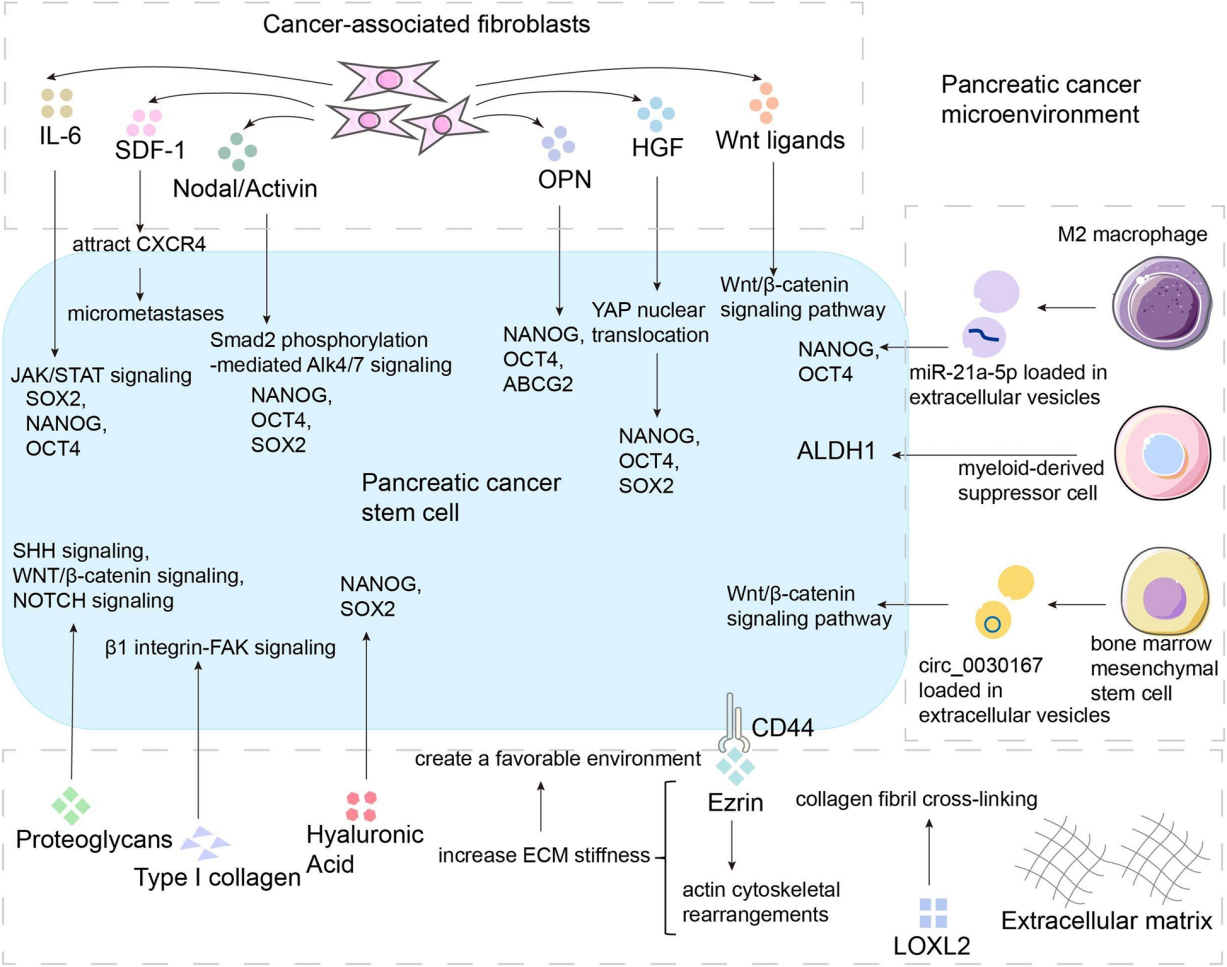
The extracellular matrix, cancer-associated fibroblasts, and immune cells are critical components in the regulation of pancreatic cancer stemness, influencing pancreatic cancer stemness-related pathways and stemness molecules through secretory factors, extracellular matrix and intercellular interactions

Developmental pathways including Sonic Hedgehog (SHH), NOTCH and WNT signaling are the most activated pathways in PC cells, which have been experimentally demonstrated to be mechanistically connected with the cancer stemness features of PC and promote PC invasion, metastasis, and drug resistance [29]. Embryonic development and stemness regulation are two fundamental mechanisms regulated by the SHH signaling pathway [30]. SHH signaling usually ceases after embryogenesis; however, the signaling pathway is reactivated during the initial progression phase of PC [31]. Additionally, studies based on RNA sequencing data suggested that compared with pancreatic ductal epithelial cells and normal pancreatic stemness, SHH and other SHH components are significantly overexpressed in CD44 + CD24 + ESA + cells, further supporting the key role of SHH in PCSCs [14]. Moreover, signal transduction of the NOTCH signaling pathway is independent of the second messenger and only occurs between cells that are in contact with each other [32]. NOTCH is activated when NOTCH receptors bind to NOTCH ligands of adjacent cells, which transmits the signals from the neighboring cell to the nucleus, starting the expression of downstream transcription factors. There are four different types of NOTCH receptors (NOTCH-1, NOTCH-2, NOTCH-3, and NOTCH-4) [33] and five kinds of NOTCH ligands (DLL-1, DLL-3, DLL-4, Jagged-1, and Jagged-2) [34]. Upregulation of several NOTCH pathway components in PCSCs has been demonstrated previously. For instance, the overexpression of Hes1 promotes PCSC self-renewal and tumorigenicity [35], and NOTCH promotes apoptotic resistance in PCSCs potentially through activation of the nuclear factor of NF-κB [36].In addition, in the TME, NOTCH signaling cascades interact with fibroblast growth factor and WNT signaling cascades to maintain cancer stemness and reshape TME [37]. WNT signaling is also essential for the maintenance of cancer stemness [38]. Aberrant activation of the canonical WNT/β-catenin signaling pathway facilitates cancer stemness renewal, thus playing vital roles in tumorigenesis and the therapeutic response of a wide range of malignancies, including PC [39]. Figures 2 and 3 shows the specific regulatory mechanisms of the three classic pathways on PCSCs.

In addition, JAK/STAT3, TGF-β, PI3K/Akt/mTOR, and Hippo signaling are also involved in the maintenance of PC stemness [40–44]. Together, these signaling pathways interact with each other and with other oncogenic signaling pathways, which provides evidence for the molecular mechanism of the PC stemness and suggests a potential approach for targeting the cancer stemness in patients with PC.

#### Targeting stemness-related pathways in PC

A promising method for targeting PCSCs is to inhibit the developmental pathways, including SHH, NOTCH, and WNT pathways, which play significant roles in maintaining and promoting PC progression by regulating PC stemness signaling [45–47].

Both SMO and the GLI family of zinc finger transcription factors in the SHH signaling pathway are regarded as important targets for cancer therapy. Cyclopamine was the first SMO inhibitor to be discovered and it can induce gemcitabine sensitivity [48, 49]. Sonidegib is a highly effective SMO inhibitor, and it has been utilized as an SHH pathway antagonist, acting by binding to SMO and inhibiting the activation of downstream hedgehog target genes [50]. α‐Mangostin has been shown to inhibit the expression of stemness-related genes CD24, CD44, CD133, NANOG, OCT4, c‐Myc, SOX2, and KLF4 by inhibiting the GLI transcription, indicating that it can regulate cancer growth by inhibiting cancer stemness population [51]. Another GLI transcription factor inhibitor GANT-61 is able to block DNA binding of GLI and decreases transcriptional activity of pluripotency-promoting factors in PCSCs, thereby reducing cancer cell growth and proliferation [52, 53].

NOTCH signaling is triggered by γ-secretase-mediated cleavage of the NOTCH receptor, and thus γ-secretase is a central player in the NOTCH signaling pathway. Studies have shown that γ- secretase inhibitors (GSI), such as MK0752, PF-03084014, and MRK-003 induce cancer cell apoptosis and interfere with cancer cell proliferation and invasion in several human cancers including PC [54–58]. In conclusion, GSI has exhibited antitumor effects in human cancer in many preclinical models; however, GSI shows a variety of side effects, such as goblet cell metaplasia of the small intestine and diarrhea [59]. Some studies have used less toxic alternative therapies, such as quinomycin, to avoid the limitations of GSIs [60]. Some natural compounds inhibiting the NOTCH signaling pathway that are non-toxic to human cells have also been identified, such as genistein, curcumin (diferuloylmethane), sulforaphane, quercetin and cimigenoside [61–66], which are expected to become therapeutic agents targeting PCSCs.

Besides, there is substantial evidence that targeting WNT/β-catenin signaling pathway enhances the sensitivity of PC to chemotherapeutic agents [67]. As previously mentioned, the WNT pathway cannot be activated in the absence of WNT ligands. WNT ligands can be palmitoylated by Porcupine (PORCN), a membrane-bound member of the o-acyltransferase family of proteins, allowing them to secret and initiate cellular reactions [68]. Several inhibitors that target PORCN prevent WNT ligand proteins from being palmitoylated in the endoplasmic reticulum, which restricts their secretion subsequently. Thus, an effective treatment strategy is to abolish WNT secretion by blocking its acylation with a PORCN inhibitor. The small molecule inhibitor WNT974 (LGK974), which is accessible orally, inhibits tumor development in vivo and decreases the viability of epithelial ovarian cancer (EOC) cells in vitro. In EOC preclinical mouse models, WNT974 exhibits improved anticancer activity in combination with paclitaxel. Currently, there is a phase I clinical trial evaluating WNT974 monotherapy for patients with PC (NCT01351103). Vantictumab (OMP-18R5), a monoclonal antibody, can specifically target FZD. Further, OMP-18R5 inhibits tumor growth in xenograft mouse models of PC and many other malignancies and is now being investigated in a phase I trial for PC (NCT02005315) [69]. There are also some agents that target the β-catenin-destruction complex. For example, an existing FDA-approved medicine, pyrvinium, can bind all CK1 family members in vitro, and selectively enhance CK1α kinase activity. By decreasing β-catenin levels and blocking the transcription of β-catenin targeted genes, pyrvinium inhibits the WNT signal. Pyrvinium reduces the development of platinum-resistant tumor and promotes apoptosis in vitro and in vivo, and when combined with paclitaxel, these effects are strengthened. However, pyrvinium no longer has an effect on cancer cells with rising levels of β-catenin [70]. A phase 1 clinical trial investigating pyrvinium for PC that cannot be removed surgically is underway (NCT05055323). In addition, many potential compounds targeting PCSCs through inhibiting the WNT/β-catenin signaling pathway have been investigated in preclinical evaluations. For example, a preclinical evaluation revealed that FH535 inhibited β-catenin transcriptional activity [71] and suppressed the expression of stemness markers CD24 and CD44 [72]. Salinomycin showed a significant inhibitory effect on increasing the cytotoxic effects of traditional therapy of gemcitabine in PCSCs [73] and inhibiting tumor cell growth and migration by interfering with LPR phosphorylation and inducing its degradation in the xenograft model in vivo [74]. Besides, some studies have also demonstrated that some natural dietary compounds, including curcumin, sulforaphane, genistein, lycopene, and piperine, can inhibit the WNT/β-catenin signaling pathway, thus inhibiting cancer stemness [75, 76] A summary of drugs targeting these pathways is provided in Table 2.

**Table 2:** Summary of drugs targeting pancreatic cancer stemness related signaling pathways

Category	Drug	Drug development stage	Treatment
Sonic Hedgehog (SHH) pathway inhibitor	Smo inhibitor		
	Cyclopamine	Preclinical	
	Sonidegib	Phase 2 (NCT02358161)	Sonidegib + Gemcitabine + Nab-paclitaxel
	Gli inhibitor		
	α‐Mangostin	Preclinical	
	GANT-61	Preclinical	
Notch pathway inhibitor	γ- secretase inhibitors (GSI)		
	MK0752	Phase 1 (NCT01098344)	Gemcitabine Hydrochloride + MK0752
	PF-0308401	Phase 2 (NCT02109445)	Gemcitabine + Nab-Paclitaxel + PF-03084014
	MRK-003	Preclinical	
	Quinomycin	Preclinical	
	Genistein	Phase 2 (NCT00376948)	Gemcitabine + Erlotinib + Genistein
	Curcumin (Diferuloylmethane)	Phase 1 (NCT02336087)	Gemcitabine Hydrochloride + Paclitaxel Albumin + Metformin Hydrochloride + a Standardized Dietary Supplement (including curcumin)
			Curcumin
			Gemcitabine + Curcumin
			Gemcitabine + Curcumin + Celebrex
		Phase 2 (NCT00094445)	
		Phase 2 (NCT00192842)	
		Phase 3 (NCT00486460)	
	Quercetin	Preclinical	
	Cimigenoside	Preclinical	WNT974 (LGK974) + PDR001
	Salinomycin	Preclinical	
	PORCN inhibitor		OMP-18R5 + Nab-Paclitaxel + Gemcitabine
Wnt/β-catenin pathway inhibitor	WNT974 (LGK974)	Phase 1 (NCT01351103)	
	FZD antagonist		
	OMP-18R5	Phase 1 (NCT02005315)	
	LRP inhibitor		pyrvinium
	Salinomycin	Preclinical	
	β-catenin-destruction complex antagonist		
	pyrvinium	Phase 1 (NCT05055323)	Genistein + Gemcitabine + Erlotinib
	β-catenin transcriptional activity inhibitor		
	FH535	Preclinical	
	Genistein	Phase 2 (NCT00376948)	
	Lycopene	Preclinical	
	Piperine	Preclinical	

Intracellular elements of tumor cells participate in the regulation of PCSC plasticity.

Many intrinsic regulators regulating PC stemness are converge into the above-mentioned stemness-related signaling pathways (WNT/β-catenin, NOTCH, SHH, JAK/STAT3, TGF-β, and Hippo). In addition, there are still many independent factors involved in the regulation of PC stemness. These factors can be categorized into several subclasses as listed in Table 3.

**Table 3:** Classification of intracellular elements of tumor cells that influence pancreatic cancer stemness plasticity

Transcription factors	PAF1, EHF, SNAI2
Epigenetic regulators	FTO, SIRT1, CRL4B, MBD3, UCHL5, GALNT3, B3GNT3, EZH2, BMI-1
Metabolic regulators	OPA1, DRP1, MYC, PGC-1α, LKB1, PGC-1β, NAF-1
Signaling pathway regulators	Tetraspanin-8, FAM83A, RER1
MicroRNAs (miRNAs)	OncomiRNAs: miR-10b, miR-17-5p, miR-21, miR-27a, miR-221, miR-338-5p, miR-520 h, miR-1246 Tumor suppressor miRNAs: miR-34a, miR-101, miR-145, miR-146a, miR-146b-3p, miR-183, miR-200a/c, miR-203, miR-429
Long non-coding RNAs (lncRNAs)	GAS5, HOTAIR, XIST, DYNC2H1-4

#### Transcription factors

In recent years, an increasing number of transcription factors that bind to the promoters of genes regulating PC stemness have been identified. RNA polymerase II-associated factor 1 (PAF1) promotes the expression of the stemness-associated genes CD44, NANOG, ABCG2, and ALDH1 [19]. ETS-homologous factor (EHF) binds to the promoter of CXCR4, thereby obstructing its transcription and resulting in altered crosstalk between PC cells and pancreatic stellate cells (PSCs) [77]. Snail family transcriptional repressor 2 (SNAI2) has been proved to promote the expression of CD44, while SNAI2 gene knockout significantly reduced the number of PCSCs, thus reducing the tumorigenicity and chemotherapy resistance of PC [78].

#### Epigenetic regulators

Fat mass and obesity-associated protein (FTO) is an RNA N6-methyladenosine demethylase. FTO depletion was shown to inhibit the spheroid formation in PC cells [79], and the absence of FTO in vitro significantly reduced the mRNA and protein expression of PCSC markers including CD44, ALDH1, SOX2, NANOG, and CD133 [79]. Overall, these results suggest that FTO is essential for spheroid formation, the maintenance of stemness marker expression, and the self-renewal potential of PC. Sirtuin 1 (SIRT1) and cullin 4B-ring E3 ligase (CRL4B) interact and cooperate as a functional unit, contributing to the epigenetic silencing of tumor suppressors, and playing an important role in regulating PCSC properties [80]. Methyl CpG binding domain 3 (MBD3) protein exhibits oncogenic effects in PC. MBD3 was proved to increase stemness markers level of OCT4, NANOG and SOX2 [81]. In addition, MBD3 binds to YAP to significantly inhibit stemness maintenance in PC cells via Hippo signaling. Ubiquitin carboxyl-terminal hydrolase isozyme L5 (UCHL5) directly deubiquitinates and stabilizes ELK3 protein to activate NOTCH-1 expression and signaling, enhancing self-renewal during PC development [82]. O-glycosyltransferases GALNT3 and B3GNT3 can promote the self-renewal of PCSCs [83]. Epigenetic regulation mediated by polycomb group (PcG) proteins, such as EZH2 and BMI-1, is also a major driver in PCSC pathogenesis [84, 85].

#### Metabolic regulators

There is growing evidence that CSC metabolism has unique characteristics. The maintenance of the PCSC phenotype is mainly related to the mitochondrial regulation of redox homeostasis and energy metabolism. Firstly, mitochondrial fusion and fission represent the main events involved in mitochondrial dynamics, and both processes are mainly controlled by different members of the dynamin family, together with several bridging proteins [86, 87]. Optic atrophy 1 (OPA1) regulates mitochondrial function and stabilizes the respiratory chain supercomplex by participating in the formation of mitochondrial cristae junctions and driving mitochondrial fusion, controlling mitochondrial respiratory activity and thereby promoting PC stemness [88]. Mitofusin-1 and mitofusin-2 are also involved in mitochondrial fusion, but the specific mechanism of their role in PCSCs has not been elucidated [86]. Besides, emerging evidence indicates that mitochondrial fission enhances uncoupled respiration to avoid excessive ROS production, thereby preventing oxidative damage. Maintaining low mitochondrial ROS levels is essential for maintaining PCSC self-renewal and function. The GTPase of the kinesin superfamily of proteins, dynamin-related protein 1 (DRP1), is the primary enforcer of mitochondrial fission [89], and PCSCs exhibit increased DRP1 expression, which is positively correlated with the expression of PC stemness-related genes such as NANOG, OCT4 and SOX2 [86]. Inhibition of mitochondrial division by inhibition of DRP1 induces the accumulation of dysfunctional mitochondria, limiting the ability of PCSCs to activate alternative pathways of energy production [86]. In addition, peroxisome proliferator-activated receptor gamma co-activator 1 (PGC-1) is the pivotal regulator of mitochondrial activity. MYC binds to the PGC-1α promoter to directly inhibit PGC-1α, thereby suppressing mitochondrial respiration and reducing PC stemness. When MYC is inhibited, the subsequent increase in PGC-1α is critical for oxidative phosphorylation in PCSCs [90]. Liver kinase B1 (LKB1) is highly expressed in CD44 + PC cells [91]. It promotes the expression of PGC-1β, which further promotes the expression of pyruvate dehydrogenase (PDH), a key enzyme linking glycolysis and the tricarboxylic acid (TCA) cycle, as well as increasing the rate of mitochondrial fusion, thereby promoting PC stemness [91, 92]. Nutrient-deprivation autophagy factor-1 (NAF-1) is highly expressed in the endoplasmic reticulum and outer mitochondrial membrane of PCSCs, and is involved in maintaining mitochondrial homeostasis to promote mitochondrial respiration, thus promoting PC stemness [93].

#### Signaling pathway regulators

In addition to ligands of the stemness-related signaling pathways in PC, some intracellular and membrane proteins are also involved in the regulation of these pathways. Tetraspanin-8 expression enhances SHH signaling [94]. Tetraspanin-8 directly interacts with PTCH, and ATXN3 is subsequently recruited into the SHH-PTCH complex, which reduce ubiquitination of PTCH and inhibits the degradation of SHH-PTCH complex mediated by proteasome. Stable SHH and PTCH promotes the binding of GRK2 protein kinase to SMO, allowing GRK2 to enhance SMO phosphorylation, relieving the repressive effects of PTCH on SMO, and leading to the activation of GLI and subsequent downstream gene expression [94]. In addition, family with sequence similarity 83 member A (FAM83A) promotes the activation of WNT/β-catenin signaling [95, 96]. In the nucleus, FAM83A binds to TCF, which in turn promotes the transcription of WNT target genes [96]. Meanwhile, FAM83A tyrosine 138 phosphorylation promoted β-catenin binding to TCF, inhibited TCF recruitment to histone deacetylases and enhanced WNT/β-catenin-mediated transcriptional and oncogenic effects [96]. In the cytoplasm, the DUF1669 structural domain of FAM83A mediated the interaction between FAM83A and AXIN1, GSK3β, and β-catenin, which in turn inhibited the phosphorylation and degradation of β-catenin protein [96]. In vivo experiments further demonstrated that FAM83A overexpression enhances tumor-initiating capacity [95]. Retention in endoplasmic reticulum 1 (RER1) has been previously shown to promote the activation of the NOTCH signaling pathway by increasing the activity of the γ-secretase complex in the brain [97]. In PC, in vitro experiments have demonstrated that RER1 promotes stemness in a hypoxic environment, including enhancing tumorsphere formation ability and stemness markers expression such as CD133, SOX2, BMI-1, Lin28, and NANOG, but the specific effect of RER1 on NOTCH signaling in PC still needs to be further explored [98].

#### Noncoding RNAs

Noncoding RNAs (ncRNAs) represent a class of RNA molecules that do not encode proteins, including from long noncoding RNAs (lncRNAs) with more than 200 nucleotides to piwi-interacting RNAs (piRNAs) with only 20 nucleotides in terms of length [99]. This type of RNA can regulate the expression of protein-coding genes; therefore, it is essential to control cell function and identity, and is related to many pathological diseases, particularly cancer [100]. Recently, ncRNAs have been shown to be involved in the regulation of stemness in different types of cancers, including PC [101].

MicroRNAs (miRNAs) regulate gene expression and maintain cell homeostasis via recognizing cognate sequences and interfering with transcriptional, translational, or epigenetic processes, and their dysregulation is associated with the regulation of PC stemness features [102, 103]. Several PC stemness factors, including NANOG, SOX2, OCT4, and ALDH1, are critical for the maintenance of PC stemness pluripotency, and miRNAs control their expression. Different types of miRNAs play different roles in regulating PC stemness. Here, we divide the regulation modes of these miRNAs on PC stemness into two categories—the enrichment of oncomiRNAs that promote PC stemness and the down-regulation of tumor suppressor miRNAs. OncomiRNAs including miR-10b [104], miR-17-5p [105], miR-21 [106, 107], miR-27a [108], miR-221 [107, 109], miR-338-5p [110], miR-520 h [111], and miR-1246 [112] have been documented. Many of these miRNA dysregulations converge on regulating stemness-related signaling pathways and the expression of cancer-relating genes. For example, miR-10b facilitates EGF-TGF-β cross-talk and enhances the expression of EMT-promoting genes, whereas decreasing the expression of several metastasis-suppressing genes [104]. MiR-17-5p can reduce the expression of tumor suppressor gene PTEN [105]. MiR-338-5p is involved in promoting WNT/β-catenin signaling patwhway [110]. Tumor suppressor miRNAs that have been reported include miR-34a [113], miR-101 [114], miR-145 [115], miR-146a [116], miR-146b-3p [117], miR-183 [118],miR-200a/c [118], miR-203 [118], miR-429 [119]. The miR-34 and miR-200 families are two main tumor-suppressive miRNA families related to regulating cancer stemness. The miR-34 family can inhibit the expression of stemness-related pathways such as NOTCH, WNT/β-catenin, TGF/SMAD and EMT-related genes such as Snail, Slug, and ZEB [120]. CD44 + and CD133 + PCSC subsets show a decrease in miR-34a expression. Restoring miR-34a expression inhibits CD44 and CD133 expression in vitro and suppresses tumor formation in vivo. In addition, miR-34a sensitizes PC cells to 5-fluorouracil (5-FU), docetaxel, and gemcitabine treatment by inhibiting NOTCH signaling [121, 122]. The miR-200 family comprises five members, including miR-200a, miR-200b, miR-200c, miR-141, and miR-429. MiR-200a overexpression was reported to reduce the expression of CD24, CD44, and ESA [123]. It was also reported that miR-200c overexpression decreases colony formation, invasion, and chemoresistance of PCSCs [124]. Treatment of PCSCs with metformin could induce the re-expression of miR-200c which is frequently lost in PC and reduce the expression of the PC stemness factors CD44, EpCAM, EZH2, NOTCH-1, NANOG, and OCT4 [125]. The reexpression of miR-101 was sufficient to limit the expression of EZH2 and EpCAM [114].

Furthermore, by competitively sequestering miRNAs, lncRNAs can act as competing endogenous RNAs (ceRNAs) together with miRNAs and mRNAs to form ceRNA networks, which can modulate the expression levels of their downstream stemness-related target genes. The overexpression of Growth arrest‐specific 5 (GAS5) which was identified as a tumor suppressor repressed the stemness features of PC cells through directly binding the 3’UTR of miR‐221 to repress its expression and increasing the expression of suppressor of cytokine signaling 3 [126]. LncRNA HOTAIR sequesters miR-34a, activating the JAK2/STAT3 signaling pathway to promote PC stemness [127]. LncRNA XIST modulates PC stemness by acting as a sponge of miR-429 [119]. Lnc-DYNC2H1-4 promotes PCSC phenotypes by sponging miR-145 [115].

These findings provide new insights for targeting PCSCs. A brief summary of these intracellular regulators is tabulated in Table 3.

#### Stimuli in the TME participate in the regulation of PCSC plasticity

A growing number of studies have shown that the complex pancreatic TME is vital in supporting stemness phenotype. The TME comprises various components. The extracellular matrix (ECM), composed of collagen, proteoglycans and glycosaminoglycans, is a major component of the TME and mediates the interaction between tumor cells and stromal cells. Cancer-associated fibroblasts (CAFs) mediate the proliferation, angiogenesis, invasion and metastasis of tumor cells. Migration and proliferation of endothelial cells lead primarily to the formation of new capillaries that support tumor progression, invasion and metastasis. Immune cells can regulate tumor activity. A classic histological feature of PC is the tumor cell-induced pro-fibrous connective tissue microenvironment, which intertwines with the ECM to provide a dense physical protective barrier for PC cells. Activation of the PSCs to an activated CAFs phenotype is accompanied by increased production of ECM components, cumulatively termed as fibrosis. In this section, we discuss the emerging knowledge about the impact of the TME on PCSCs.

#### ECM

ECM is a highly dynamic structural component. In PC models, abnormal collagen cross-linking generates mechanical stress and increases ECM stiffness, which can create a favorable environment for PCSC survival and thus enhance their viability.

LOXL2 promotes collagen fibril cross-linking leading to ECM remodeling, thereby promoting PC stemness [128]. CD44 interacts with ezrin in the TME to regulate actin cytoskeletal rearrangements to promote PC stemness, and small molecule inhibitors of ezrin have been shown to reduce the self-renewal capacity of PCSCs [129]. The hyaluronic acid (HA)/CD44 axis creates a suitable ecological niche for PCSC survival by increasing centrosome abnormalities and micronucleation, as evidenced by increased expression of NANOG and SOX2 [130]. HA can also bind toll-like receptor 2 and 4 to promote inflammatory gene expression and exacerbate the inflammatory response at the tumor site. Subsequently, cytokines and inflammatory mediators secreted by tumor-associated immunosuppressive cells contribute to PC stemness [131, 132]. The most plentiful ECM protein, type I collagen, is the main scaffold for CD133 + and ALDH + PCSCs and increases PCSC enrichment by activating β-integrin and FAK [133]. Type I collagen activates β-integrin, which is required for FAK activation [133, 134]. The tyrosine protein kinase FAK is recruited by the activated β1-integrin to initiate signal transduction after activation [134]. The FAK domain autophosphorylates the Tyr 397 residue in response to integrin engagement, enlisting SRC family kinases, and then phosphorylates the Y576 and Y577 residues in the catalytic domain [134]. This represents the initial and major step in FAK activation, which further promotes ALDH1 expression. ECM can regulate the PCSC niche by creating a hypoxic environment that directly activates HIF and target genes, as well as regulate the cascade of stemness factors and pathways, such as OCT4 and NOTCH, and enhance the expression of stemness markers [135, 136]. Proteoglycans combine with various cytokines and chemokines in the TME to activate various signaling pathways in PCSCs, such as SHH, WNT/β-catenin, and NOTCH. For example, glypican-4 (GPC4) is a member of proteoglycans, which enhances stem cell–like properties via promotion of WNT/β-catenin pathway and decrease the sensitivity of PC cells to 5-FU [137].

#### CAFs

In the pancreatic TME, cancer-associated fibroblasts (CAFs) are abundant stromal-activated fibroblast cell types that can regulate PC stemness and are functionally important in tumor development and metastasis [138]. PC cells are capable of secreting different levels of signaling molecules, such as TGF-β, SHH, IL-6, and TNF-α, and then activating CAFs [139]. Activated CAFs, in return, release growth factors, chemokines or cytokines to directly affect cancer cells. CAFs form a paracrine niche for PCSCs, wherein paracrine signaling enhances PC stemness-like properties at the tumor-stroma interface [140]. In this section, we illustrate the effect of some cytokines secreted by CAFs on PC stemness.

A recent report suggested that the osteopontin (OPN) released by CAFs was regarded as a crucial driver of PC development by upregulating the plasticity of PCSCs [141]. OPN, a multifunctional secreted integrin-binding glycoprotein, is overexpressed in numerous cancers and can be identified as a prognostic factor clinically [142, 143]. OPN in the tumor microenvironment binds to CD44 expressed on PCSC properties, which subsequently promotes clonal growth, invasion, and metastasis. These effects require CD44 to bind to the protein encoded by the oncogene TIAM1, which activates Rac1 to induce membrane cytoskeleton-mediated cell adhesion, proliferation, and migration. Previous studies have shown that ovarian cancer cells stimulated mesothelial cells to promote OPN expression and release through TGF-β signaling. OPN promoted ovarian cancer stemness and chemoresistance via PI3K/AKT signaling, CD44 receptor activation, and ABC drug efflux transporter activity [144]. In addition, the glioma perivascular niche facilitates stemness characteristics via the OPN–CD44 signaling pathway was also demonstrated [145]. CAFs promotes PC stemness through the interaction between OPN–CD44 axis and tumor cells has also been confirmed [141]. The OPN secreted by CAFs acts on the CD44 receptor of PCSCs and promotes the stemness characteristics of PC by promoting the expression of stemness markers NANOG, OCT4, and ABCG2 [141].

Furthermore, hepatocyte growth factor (HGF), which is associated with the regulation of PC stemness, is also secreted by CAFs. When HGF binds to c-MET in cancer cells, they are stimulated to produce uPA, causing more pro-HGF to become active HGF, which then binds to c-MET in PC cells. Paracrine HGF induces YAP nuclear translocation by binding c-MET, resulting in the crosstalk between CAFs and cancer cells that HGF/c-MET-mediated induces the expression of stemness pluripotency markers such as NANOG, OCT4, and SOX2 in PC cells, as well as increased self-renewal ability [146]. In addition, YAP nuclear translocation is followed by binding to HIF-1α in the nucleus and maintains the stability of HIF-1α to promote glycolysis [147]. Glycolysis is a key characteristic of both normal stemness and cancer stemness, forming a novel metabostem property [148]. Even under aerobic conditions, tumor cells favor a metabolic transition to glycolysis, and this metabolic reprogramming is crucial in the development, maintenance, and differentiation of cancer stemness and is a hallmark of cancer stemness [148]. Cancer stemness is more dependent on glycolysis for bioenergy during the metabolic shift. Increased expression and stability of HIF-1α promote this metabolic reprogramming process [149]. Therefore, HIF is critical for promoting the stemness of PC. On the one hand, HIF-1α enhances the expression of hexokinase 2 **(**HK2), one of the rate-limiting enzymes in the glycolytic pathway [150]. On the other hand, HIF-1 directly stimulates the expression of c-MET. HIF-1α promotes c-MET signaling by inducing c-MET gene expression in response to metabolic stress, such as nutrient deficiency or hypoxia, thereby promoting PC stemness [151].

In addition, CAFs secrete the ligands of WNT and promotes PC stemness through the WNT/β- catenin linked protein pathway [152, 153]. IL-6 secreted by CAFs upregulates the expression of PCSC genes, including SOX2, NANOG, and OCT4 by activating the JAK2/STAT3 pathway [154]. CAFs also produce SDF-1, the ligand for CXCR4, to attract CXCR4 + PCSCs, causing micrometastases [155]. Nodal/Activin secreted by CAFs acts on Alk4/7 receptors on PCSCs to promote self-renewal [156]**.** Thus, CAFs play an important role in propagating PC stemness phenotype.

#### Immune cells and extracellular vesicles

The enhanced ability of PCSCs to promote tumor development suggests that these cells have an innate advantage for immune escape. The role of immune cells against tumors is two-fold, with both tumor-promoting and tumor-suppressing activities. Evidence is accumulating that several immune cell types have an important role in regulating PCSC properties. For example, in the context of PC cells, monocytes acquire an immunosuppressive phenotype by activating STAT3 and become myeloid-derived suppressor cells, while this STAT3 activation promotes ALDH1 + stem cell frequency in PC [157]. MiR-21a-5p is upregulation in M2 macrophage-derived extracellular vesicles (EVs), which promotes NANOG and OCT4 expression and sphere-forming, colony-forming, invasion, migration, and anti-apoptosis abilities of PCSCs in vitro and tumorigenic ability in vivo [158]. Targeting tumor-infiltrating macrophages decreased the number of PCSCs, relieved immunosuppression and improved chemotherapeutic responses in PC [159]. Circ_0030167 loaded in EVs derived from bone marrow mesenchymal stem cells inhibits the stemness of PC cells by sponging miR-338-5p and further regulating the WNT/β-catenin axis [110].

#### Metabolic plasticity of PCSCs and the impact of epigenetic regulation on it

Besides RNAs, cytokines, morphogens and growth factors, various metabolic pathways are also involved in stemness destiny control. Metabolic pathways can also transmit changing signals in the extrinsic environment to alter intrinsic cell fate. PCSC metabolism represents a combination and balance of intrinsic metabolic demands and extrinsic metabolic alterations. In this section, we describe the metabolic plasticity of PCSCs.

PCSCs are metabolised in a different way to other PC cells. For example, non-PCSCs are highly dependent on glycolytic metabolism, whereas PCSCs are strongly dependent on the mitochondrial oxidative phosphorylation (OXPHOS) pathway. This metabolic reprogramming of PCSCs is also plastic and can be regulated by the environment in which they are located. The use of the more energetically efficient metabolic pathway—OXPHOS in the presence of sufficient oxygen results in a higher number of ATP molecules per glucose molecule. Under hypoxia or stress, these stemness can revert to a glycolytic program, even in some cases using mitochondrial fatty acid oxidation. For example, when the mitochondrial inhibitor metformin was used in PC, metformin-resistant PCSCs reversed towards a non-stemness metabolic phenotype by increasing MYC expression to enhance glycolytic capacity [90]. Thus, disruption of mitochondrial metabolic dynamics is likely to attenuate the stemness phenotype of PC. Besides, in the process of dedifferentiation of PC cells, cells increase their oxidative metabolism by promoting pyruvate to enter the TCA cycle and improving the expression levels of citrate and citrate lyase in cells. The rapid transformation of this metabolic change indicates that it is closely related to epigenetics.

Mitochondrial dynamics and metabolism are mainly controlled by post-translational modifications (PTMs) of proteins, among which ubiquitination (Ub) and Ub-like (UbL) modifiers plays a major role in the regulation of PC stemness. This is very similar to the regulatory mechanism in normal stemness, where the ubiquitination and deubiquitination activities of the stemness pathways NOTCH, WNT, and SHH proteins are precisely regulated by ubiquitinating and deubiquitinating enzymes, resulting in the reprogramming of PC stemness metabolism. Among the UbL modifiers, the expression of interferon-stimulated gene 15 (ISG15), small ubiquitin-related modifier (SUMO), neural precursor cell expressed developmentally downregulated protein 8 (NEDD8), and human leukocyte antigen-F adjacent transcript 10 (FAT10) are significantly higher in CD133 + cells than in CD133- cells. A recent study showed that ISG15 and protein ISGylation are specifically enriched in PCSCs compared with non-PCSCs. Loss of ISG15/ISGylation alters the mitochondrial state and metabolism—manifested by an increase in the number of mitochondria but a severely impaired optical character recognition, that is, the accumulation of dysfunctional mitochondria. In addition, the glycolytic capacity of PCSCs is also significantly impaired in the absence of ISG15, indicating that the overall metabolic plasticity of PCSCs (aerobic and anaerobic respiration) is affected by the loss of ISG15/ISGylation. However, the regulation of OXPHOS by SUMO, NEDD8, and FAT10 in PCSCs has not been reported yet.

Besides, glutamine is a major mitochondrial reaction substrate and is also required for the maintenance of mitochondrial membrane potential and integrity. In the CD9 + PC stemness subpopulation, CD9 increases glutamine uptake and promotes mitochondrial OXPHOS by interacting with the glutamine transporter ASCT2 [160].

### PCSCs and malignant phenotype

#### PCSCs and EMT promote each other significantrelationship

A crucial developmental program called EMT is frequently engaged during the invasion and metastasis of cancer. It is of fundamental importance in biology that the activation of the EMT process is related to the characteristics of stemness in neoplastic cells [161, 162]. During the shifts towards the mesenchymal phenotype, which represents a more invasive and aggressive disease phenotype, levels of non-invasive epithelial cell markers, including E-cadherin, α-catenin, and γ-catenin decrease, while the expression of vimentin, metalloproteinases MMP-2, MMP-9, fibronectin, and N-cadherin which are typically expressed on invasive epithelial cells increase [163]. A group of transcription factors called EMT-activating transcription factors, including Snail, Slug, Twist1, NF-κB, ZEB1, and ZEB2 control EMT by suppressing the expression of genes that code for epithelial markers, such as E-cadherin. ZEB1 is the most crucial promoter of EMT and links EMT with stemness-maintenance in PC as shown in the K-ras^LSLG12D/+^; Trp53^R172H/+^; Pdx-1-Cre (KPC) mouse models [164]. As a transcriptional repressor, ZEB1 binds to the E-box motif in the promoter regions of downstream target genes, such as E-cadherin and members of the miR-200 family, to decrease their production. It is also demonstrated that ZEB1 and stemness marker CD44 are mutually regulated. ZEB1 suppresses the epithelial splicing regulator ESRP1 in PC to promote CD44 isoforms (CD44s) splicing, causing the expression to change from the variant CD44v to the standard CD44s subtype. CD44 contains two isoforms, each with a distinct function: standard isoform (CD44s) and variant isoforms (CD44v). CD44s has been proven to be positively related with stemness gene features, whereas CD44v exhibits an inverse association [165]. Additionally, CD44s also contributes to PC lymph node and liver metastasis and advanced TNM staging [166]. An increase in CD44s level was shown to increase the expression of ZEB1, thus forming a positive feedback loop, leading to a self-sustaining ZEB1 and CD44s expression. The feedback loop between CD44s and ZEB1 influences the ability of cancer cells, including the increase of tumorsphere initiation and metastasis ability [167]. In addition, nestin, one of the PC stemness markers is also vital in PC cell metastasis, and the administration of nestin siRNA was reported to provide a novel therapeutic strategy for PC [168]. Nestin, NANOG, Slug, and MMP2 mRNA levels decreased, and E-cadherin expression levels increased in nestin shRNA-transfected PC cells [169]. An orthotopic implantation model using mice also showed that nestin knockdown significantly reduced primary and metastatic tumor development by human PC cells [168]. Besides, the expression of PC stemness marker CD133 reportedly induces EMT via the transcription factor NF-κB [170, 171]. Compared with CD133- cells, CD133 + cells showed increased NF-κB expression [172]. Mechanically, the overexpression of CD133 increases both mRNA and protein levels of IL-1β gene expression, and then IL-1β activates NF-κB, thus driving EMT and cell invasion [173].

#### Therapeutic resistance

Recently, a growing number of studies have revealed that the potential etiology of therapeutic resistance is related to stemness markers of PC. Herman et al. found that CD133 + cells were more resistant to gemcitabine than CD133 − cells isolated from PC patients, and prolonged exposure resulted in the selection of CD133 + cells [26]. Furthermore, they demonstrated in a xenograft model that animals given gemcitabine experienced a reduction in tumor size but an increase in the percentage of CD133 + cells. Several research exploring the molecular interaction between therapeutic resistance and CD133 + PC cells have highlighted the critical role of its metabolic plasticity, which is related to reactive oxygen species (ROS) [174]. Low levels of ROS are critical for maintaining cancer stemness and their resistance to therapy; however, the ROS regulating mechanisms in cancer stemness remain to be explored [175]. Studies have shown that ROS production is indeed lower in CD133 + cells compared with CD133- cells. When drugs typically associated with ROS production, such as gemcitabine, 5FU, and paclitaxel, were applied to CD133 + and CD133- cells, CD133 + stemness did not exhibit any increase of ROS, while CD133- cells had enhanced ROS generation [174]. These treatments further caused CD133- cancer cells to die whereas CD133 + cells were unaffected, which seems to provide CD133 + stemness a survival advantage [174]. In addition, although gemcitabine, a cytotoxic agent, can inhibit CD133 + stemness proliferation, it has little effect on the apoptosis of cancer stemness, allowing them to return to the stemness pool upon gemcitabine withdrawal [176]. In contrast, under gemcitabine treatment, the vast majority of the tumor cells — more differentiated cells — became apoptotic [177]. It is clear that traditional therapy can only target highly differentiated tumor cells, leaving undifferentiated cancer stemness resistant to therapy.

Other stemness markers are also associated with therapeutic resistance. It is reported that CD44 + PC stemness properties show higher malignancy and stronger resistance to chemotherapy and radiotherapy than CD44- cells [178–180]. Studies have also shown that PC with high CD44 expression is resistant to gemcitabine. Knocking out CD44 also leads to decreased invasiveness and increased sensitivity to gemcitabine [178, 181]. The influence of CD44 + stemness on therapeutic-resistance is mainly attributed to the ABC superfamily of transporter proteins in PC [182]. The overexpression of ABC superfamily of transporter proteins in PC limits the exposure to anticancer drugs. CD44 is also associated with the increased protein expression of the ABC transporter genes MDR1 and MRP1 [183–185]. Verapamil, an ABC transporter inhibitor, resensitized resistant cells to gemcitabine in a dose-dependent manner, and CD44 RNA interference inhibited the clonogenic activity of resistant cells [181].

#### Clinical perspective—Detection and prediction of prognosis in patients with PC via stemness properties

The analysis of PC stemness in surgical tissue specimens is anticipated to discover meaningful and reliable prognostic indicators and evaluate the effectiveness of anticancer therapy. Recently, agrin, an extracellular matrix protein, was reported to be enriched in the extracellular vesicles of PC stemness and act as a marker of poor prognosis in patients with PC [186]. In hepatocellular carcinoma, agrin promotes hepatocarcinogenesis by binding to the LRP-4 receptor and activating the YAP transcription factor [187]. Researchers have also verified the existence of this pathway in PC cells, proving that agrin in cancer stemness extracellular vesicles promotes YAP activation and cancer cell proliferation and inventory. It can translocate YAP, the core participant of the Hippo pathway, to the nucleus in order to alter the transcriptional program of cells, thus promoting tumor proliferation and metastasis [186]. In addition, researchers found that circulating agrin + extracellular vesicles (EVs) can be used as specific and sensitive biomarkers of disease progression in patients with PC, who did not undergo surgery, through the ROC curve analysis, and combination with CD133 + EVs improves the accuracy of disease progression prediction [186]. In addition, the expression of cancer stemness markers such as CD24, CD44, and CD133 has been linked to decreased survival in PC. For example, CD24 is overexpressed in high-grade tumors and more advanced PC stages, and lymphatic invasion and venous invasion are observed more frequently in the CD24 + PC, suggesting its role in the progression of PC [188]. CD24 is related to the recurrence of resectable PC and is an important factor that leads to a low survival rate in patients with PC [189]. Overexpression of CD44 or CD133 is significantly associated with clinical TNM stage, tumor differentiation, lymph node metastasis, and a decreased 5-year overall survival rate [190, 191].

## Discussion

PC is a highly malignant tumor with a poor prognosis. Despite advancements in the treatment, late-stage diagnosis and other reasons result in poor prognosis, recurrence, and metastasis. Cancer stemness refers to the ability of a pool of self-sustaining cells in generating differentiated cancer cells and initiating tumor growth [176]. The term cancer stemness does not denote the origin, but rather the plasticity state of cancer cells. Increasing evidence supports the idea that cancer stemness exists in a highly plastic state rather than an absolute entity [192–194]. Cancer stemness have the ability to self-renew and regenerate, therefore, these cells are significantly resistant to chemo- and radiotherapy.

On the one hand, investigating cancer stemness from the standpoint of fundamental science helps improve the comprehension of tumor heterogeneity. On the other hand, refining our understanding of the plasticity of PCSCs may eventually lead to a better understanding of the clinical prospects of targeting PC stemness. In particular, targeting PC stemness has potential benefits for patients with PC. PC stemness heavily contributes to therapeutic resistance [195]. Accumulating evidence suggests that the combination of chemotherapy drugs and PC stemness inhibitors is more effective than monotherapy in vitro and in vivo [53, 196]. The relationship between PC stemness and tumor malignant phenotype demonstrates a new possibility of PC treatment based on PC stemness-targeting since PC stemness promotes tumor growth and metastasis [180]. Referring to the current state of research on PC stemness, there are several aspects in oncology that deserve further study. It is obvious that targeting PC stemness should be an integral part of the entire treatment scheme. Targeting PC stemness, however, provides substantial hurdles since therapy regimens may damage normal stemness in the human body. The challenge ahead is to specifically target PC stemness without unnecessarily affecting normal stemness. Thus, identifying of cancer stemness-specific signaling networks is critical for the improvement of anti-stemness cancer therapy [197]. Moreover, some studies have shown that the metabolism of cancer stemness rapidly transit under heterogeneous environmental circumstances. Identifying specific metabolic pathways, such as hypoxia, nutrient deficiency, and the low pH of cancer stemness may also be beneficial [198]. Another aspect worth exploring further is the characterization of markers that can identify circulating PCSCs in liquid biopsies for PC diagnosis, prediction of prognosis and assessment of treatment response.

In conclusion, a better understanding of PC stemness and its plasticity may provide crucial insights into novel and effective treatments and improve the prognosis of patients with PC.

## Data Availability

Not applicable.

## Abbreviations

PC: Pancreatic cancer
PCSCs: Pancreatic cancer stem cells
TME: Tumor microenvironment
CSCs: Cancer stem cells
TICs: Tumor-initiating cells
ABC: ATP-binding cassette
SHH: Sonic Hedgehog
GSI: γ- Secretase inhibitors
PORCN: Porcupine
EOC: Epithelial ovarian cancer
PAF1: RNA polymerase II-associated factor 1
EHF: ETS-homologous factor
PSCs: Pancreatic stellate cells
SNAI: Snail family transcriptional repressor 2
FTO: Fat mass and obesity-associated protein
SIRT1: Sirtuin 1
CRL4B: Cullin 4B-ring E3 ligase
MBD3: Methyl CpG binding domain 3
UCHL5: Ubiquitin carboxyl-terminal hydrolase isozyme L5
PcG: Polycomb group
OPA1: Optic atrophy 1
DRP1: Dynamin-related protein 1
PGC-1: Proliferator-activated receptor gamma co-activator 1
LKB1: Liver kinase B1
PDH: Pyruvate dehydrogenase
TCA: Tricarboxylic acid
NAF-1: Nutrient-deprivation autophagy factor-1
FAM83A: Family with sequence similarity 83 member A
RER1: Retention in endoplasmic reticulum 1
ncRNAs: Noncoding RNAs
lncRNAs: Long noncoding RNAs
piRNAs: Piwi-interacting RNAs
miRNAs: MicroRNAs
ceRNAs: Competing endogenous RNAs
GAS5: Growth arrest‐specific 5
ECM: Extracellular matrix
CAFs: Cancer-associated fibroblasts
HA: Hyaluronic acid
GPC4: Glypican-4
OPN: Osteopontin
HGF: Hepatocyte growth factor
HK2: Hexokinase 2
EVs: Extracellular vesicles
OXPHOS: Oxidative phosphorylation
PTMs: Post-translational modifications
Ub: Ubiquitination
UbL: Ub-like
ISG15: Interferon-stimulated gene 15
SUMO: Small ubiquitin-related modifier
NEDD8: Neural precursor cell expressed developmentally downregulated protein 8
FAT10: F adjacent transcript 10
ROS: Reactive oxygen species
